# MicroRNA-122-5p inhibits cell proliferation, migration and invasion by targeting CCNG1 in pancreatic ductal adenocarcinoma

**DOI:** 10.1186/s12935-020-01185-z

**Published:** 2020-03-30

**Authors:** Chen Dai, Yan Zhang, Zhihua Xu, Mengxian Jin

**Affiliations:** 1 Department of Endocrinology, Suzhou Xiangcheng People’s Hospital, Suzhou, 215131 China; 2 Department of General Surgery, The First Affiliated Hospital of Soochow University, Suzhou, 215006 China; 3 Department of Oncology, The First Affiliated Hospital of Soochow University, Suzhou, 215006 China

**Keywords:** PDAC, MiR-122-5p, CCNG1

## Abstract

**Background:**

Pancreatic ductal adenocarcinoma (PDAC) is a lethal human malignancy, and previous researches support the contribution of microRNA (miRNA) to cancer progression. MiR-122-5p is reported to participate in the regulation of various cancers, while the function of miR-122-5p in PDAC remains unclear. In this study, we investigated the precise mechanism of miR-122-5p involved in PDAC pathogenesis.

**Methods:**

The expression levels of miR-122-5p were detected in human PDAC tissues and cell lines by miRNA RT-PCR. The effects of miR-122-5p on cell proliferation were explored by MTT assays, colony formation assays and flow cytometry assays. The ability of migration and invasion was determined by transwell assays. Dual Luciferase reporter assay was performed to validate the direct interaction between miR-122-5p and its target gene. The related molecules of cell cycle, apoptosis and epithelial–mesenchymal transition (EMT) were examined with qRT-PCR and western blot. In addition, xenograft mouse models were applied to explore the effects of miR-122-5p in vivo.

**Results:**

MiR-122-5p was underexpressed, while CCNG1 was highly expressed in PDAC tissues and cells. MiR-122-5p was negatively correlated with TNM stage, tumor size and lymph node metastasis in PDAC patients. Overexpression of miR-122-5p suppressed the proliferation, migration and invasion in vitro and inhibited tumorigenesis in vivo. Furthermore, CCNG1 was a direct target of miR-122-5p. Upregulated CCNG1 could partially reverse the effects caused by miR-122-5p. Moreover, miR-122-5p inhibited EMT through downregulation of CCNG1.

**Conclusion:**

Overexpression of miR-122-5p could inhibit cell proliferation, migration, invasion, and EMT by downregulating CCNG1 in PDAC, suggesting a potential therapeutic target for PDAC.

## Background

Pancreatic ductal adenocarcinoma (PDAC) is a lethal human malignancy and predicted to be the second leading cause of cancer-related death by 2030 [1]. With a 5-year survival rate of 9%, it is usually detected at advanced stages and has limited therapeutic options [2]. PDAC exhibits the characteristics of extensive desmoplasia, rapid metastasis, and advanced resistance to chemotherapy and radiation, rendering this disease a major priority for public health care [3]. One of the main reasons for such poor prognosis is the limited understanding of molecular mechanisms of PDAC progression. Therefore, a better understanding of key mechanisms driving initiation and development of PDAC is urgently needed.

Recently, microRNAs (miRNAs), an evolutionarily conserved kind of small non-coding RNAs (sncRNAs) with a length of 18–24 nucleotides, have emerged as pivotal regulators of gene expression by directly binding to the 3′-untranslated region (UTR) of the targeted genes [4]. MicroRNAs have been reported to be involved in a variety of cellular processes, including differentiation, proliferation, apoptosis, invasion and migration [5, 6]. Dysregulated miRNAs have been correlated with tumorigenesis, cancer progression and response to therapy with a role that activates or silences some oncogenes or tumor suppressors in human [7, 8]. As reported, an alteration in miRNA expression has contributed a lot in a variety of cancers [9]. Mechanisms underlying the interactions between miRNAs and corresponding targeted mRNAs in cancer are under urgent priority to be elucidated.

In a large family of miRNAs, miR-122-5p was first shown to play a role in breast cancer by targeting ADAM10 [10]. Later, other studies indicated that miR-122-5p could act as a tumor suppressor in hepatocellular carcinoma [11], renal cell carcinoma [12], gastric cancer [13], bile duct carcinoma [14] and so on. In 2017, Calatayud et al. found that miR-122-5p was downregulated in pancreatic cancer tissues compared to that in paired normal tissues [15]. A same conclusion appeared in Zhou’s research [16]. However, the specific molecular mechanism of miR-122-5p in the field of PDAC is still unclear. In the present study, we tended to elucidate the role of miR-122-5p in PDAC. We found that miR-122-5p was downregulated in both pancreatic cancer tissues and cells, and overexpression of miR-122-5p could inhibit cell proliferation, migration and invasion of PDAC.

Through bioinformatic prediction and experimental confirmation, cyclin G1 (CCNG1) was identified as the putative target of miR-122-5p. CCNG1 was firstly found in 1996, located on chromosome 5q-32-q34 with six exons at length [17]. It is a subtype of Cyclin G of the cyclin family which positively or negatively regulates cell proliferation through cell cycle in different kinds of cancers [18, 19]. Former researches focused on CCNG1 have suggested lots of valuable conclusions. Han et al. discovered that CCNG1 was upregulated in lung cancer tissues and cells and presented a new target for treatment of lung cancer [20]. A study of Zhao et al. indicated that CCNG1 could promote tumorigenesis and epithelial-mesenchymal transition (EMT), which influenced cell proliferation, migration and invasion of esophageal carcinoma [21]. However, the studies of CCNG1 in PDAC have not been clarified. In our research, we demonstrated that CCNG1, which could be directly regulated by miR-122-5p, was overexpressed in pancreatic cancer tissues and cell lines, and could antagonize the effects caused by miR-122-5p in inhibiting cell growth, migration and invasion.

In conclusion, we explored the functions of miR-122-5p and its correlation with CCNG1 in PDAC. Our results suggested that the downregulation of miR-122-5p had vital effects in affecting cell proliferation, migration, invasion and EMT of PDAC, presenting miR-122-5p the role as a novel therapeutic target for treatment of PDAC.

## Materials and methods

### Cell lines and cell culture

Two human pancreatic adenocarcinoma cell lines (PANC-1 and PL-45) used in this study were purchased from the Chinese Academy of Sciences Cell Bank (Shanghai, China). The cells were cultured in RPMI-1640 (Gibco, Thermo Fisher Scientific Inc., Waltham, MA) medium containing 10% fetal bovine serum (Gibco, Thermo Fisher Scientific Inc., Waltham, MA), 100 units/mL penicillin and 100 μg/mL streptomycin and incubated at 37 °C in a humidified chamber with 5% CO_2_.

### Patients and tissue samples

Human primary pancreatic cancer tissues and paired normal adjacent tissues were obtained from 60 patients who underwent surgical resection in the Department of Surgery, First Affiliated Hospital of Soochow University, China, from October 2015 to October 2017. All patients were diagnosed with PDAC based on histopathological evaluation. None of the patients had received chemotherapy, radiotherapy or any other anticancer therapy prior to surgery. Written informed consent was signed before specimen collection. This study was approved by the Ethics Committee of the First Affiliated Hospital of Soochow University. Tissue fragments including PDAC samples and adjacent normal tissues were frozen in liquid nitrogen right after the resection and stored at − 80 °C until RNA extraction.

The 60 samples were detected for their miR-122-5p expression using miRNA RT-PCR, indicating 27 relatively high expressed and 33 low. In total, there were 35 male and 25 female patients. These cohorts consisted of 26 patients who were under 60 years old and 34 who were over 60 years old with an average age of 62.17 ± 16.35 years. According to the TNM staging criteria issued by the American Joint Committee on Cancer (AJCC), TNM stage was determined. There were 15 patients in stage I–II and 45 in stage III–IV. 24 patients did not have lymph node metastasis, and 36 patients did. 14 patients were well-differentiated, 22 presented with moderate-differentiated carcinoma, and 24 were poorly differentiated. There were 44 patients with tumor size < 5 and 16 patients with tumor size ≥ 5. Among these samples, CCNG1 was detected using qRT-PCR. The results revealed that 33 patients were CCNG1 high level and 27 were CCNG1 low. The clinical information of patients was summarized in Table 1.

**Table 1:** Correlation between miR-122-5p expression and clinicopathological features in 60 PDAC patients

Variables	No. of cases (n = 60)	MiR-122-5p expression		P-value
		Low (n = 33)	High (n = 27)	
Gender				0.895
Male	35	19	16	
Female	25	14	11	
Age				0.875
< 60	26	14	12	
≥ 60	34	19	15	
TNM stage				0.011
I–II	15	4	11	
III–IV	45	29	16	
Tumor size				0.014
< 5	44	20	24	
≥ 5	16	13	3	
Lymph node metastasis				0.006
Yes	36	25	11	
No	24	8	16	
Differentiation				0.082
High	14	9	5	
Moderate	22	15	7	
Low	24	9	15	
CCNG1				0.011
Positive	33	23	10	
Negative	27	10	17	

*PDAC* pancreatic ductal adenocarcinoma, *TNM* tumor-node-metastasis

### MTT assay

Cell proliferation was evaluated using 3-(4, 5-dymethyl-2-thiazolyl)-2, 5-diphenyl-2H-tetrazolium bromide (MTT) assay. Cells were seeded in 96-well plates at a concentration of 3000 cells/well and cultured for 24, 48, or 72 h after transfection. After that, cells were incubated for another 4 h with a volume of 20 μL of MTT (5 mg/mL) solution per well. The medium was then replaced with 100 μL dimethyl-sulfoxide (DMSO) and vortexed for 10 min. The optimal density (OD) was read at a wavelength of 490 nm on a Versamax microplate reader (Molecular Devices, Sunnyvale, CA, USA). Blank wells without cells were designated as controls.

### Western blot analysis

Cell lines (PANC-1 and PL-45) after transfection were harvested and lysed using radio immunoprecipitation assay lysate (RIPA, PS0013, Beijing Leagene Biotechnology Co., Ltd., Beijing, China) supplemented with protease inhibitor cocktail and phosphatase inhibitor cocktail (Roche). The extracted proteins were added to sample loading buffer, boiled for ∈10 min at 95 °C, separated by 10% sodium dodecyl sulfate polyacrylamide gel electrophoresis (SDS-PAGE), and transferred to nitrocellulose membranes (Millipore, MA, USA) by semi-dry blotting. The membranes were blocked with 5% bovine serum albumin for 1 h at room temperature and incubated in primary antibody overnight at 4 °C. The primary antibodies used in this study were raised against CCNG1 (1:1000, WH0000900M1, Sigma-Aldrich), Cyclin E (1:1000, #20808, Cell Signaling Technology), Cyclin D1 (1:1000, #2922, Cell Signaling Technology), Cyclin A2 (1:1000, #91500, Cell Signaling Technology), Cyclin B1 (1:1000, #12231, Cell Signaling Technology), CDK2 (1:1500, ab32147, Abcam), CDK4 (1:1000, #12790, Cell Signaling Technology), CDK6 (1:1000, #13331, Cell Signaling Technology), P21 (1:1000, #2947, Cell Signaling Technology), P27 (1:1000, #3686, Cell Signaling Technology), Bax (1:1000, #5023, Cell Signaling Technology), Bcl-2 (1:1000, #3498, Cell Signaling Technology), E-cadherin (1:1000, #3195, Cell Signaling Technology), Vimentin (1:1000, #5741, Cell Signaling Technology), N-cadherin (1:1000, #13116, Cell Signaling Technology), MMP9 (1:1000, #15561, Cell Signaling Technology) and GAPDH (1:1000, #5174, Cell Signaling Technology). Then, the membranes were incubated at room temperature for 1 h with goat immunoglobulin G (IgG, 1:5000, ab6721, Abcam) containing conjugated horseradish peroxidase. Immunoreactive signals were developed with ECL kit (Thermo Scientific, Waltham, MA). The band density was normalized to GAPDH, and quantified by ImageJ software.

### RNA extraction and quantitative reverse transcription real-time polymerase chain reaction (RT-PCR)

Total RNA or miRNA was isolated and extracted by TRIzol reagent (Invitrogen; Thermo Fisher Scientific, Inc.) or miRcute Extraction and Separation of miRNAs kit (Tiangen Biotech Co., Ltd., Beijing, China), and then reversely transcribed into cDNA using PrimeScript™ II 1st strand cDNA synthesis kit (Takara Biotechnology Co., Ltd., Dalian, China). SYBR Premix kit or SYBR PrimeScript miRNA RT-PCR kit (both from Takara Biotechnology Co., Ltd.) was used for qRT-PCR. The thermocycling conditions were one cycle of initial denaturation at 95 °C for 3 min, 40 cycles of 95 °C for 15 s and 60 °C for 30 s. Glyceraldehyde-3-phosphate or dehydrogenase (GAPDH) and U6 small nuclear RNA (U6) were used for normalization. The relative expression levels of miRNA and mRNA between the experimental group and the control group were calculated using 2-ΔΔCq method. The experiments were repeated at least 3 times. The primers were as follows: miR-122-5p forward, 5′-TATTCGCACTGGATACGACACAAAC-3′ and reverse, 5′-GCCCGTGGAGTGTGACAATGGT-3′; U6 forward, 5′-GCTTCGGCAGCACATATACTAAAAT-3′ and reverse, 5′-CGCTTCACGAATTTGCGTGTCAT-3′; CCNG1 forward, 5′-GTTACCGCTGAGGAGCTGCAGTC-3′ and reverse, 5′-GCAGCCATCCTGGATGGATTCAG-3′; GAPDH forward, 5′-GGTGAAGGTCGGAGTCAACG-3′ and reverse, 5′-CAAAGTTGTCATGGATGHACC -3′.

### Colony formation assay

1 × 10^3^ cells were seeded into 6-well plates. During colony growth, culture medium was replaced every 3 days. The cells were stained with Crystal Violet Staining Solution (Beyotime, Shanghai, China) 10 days later, and the colony number in each well was counted.

### Flow cytometry analysis

Propidium iodide (PI) staining flow cytometry was performed for cell cycle distribution with a Cell Cycle Detection Kit (Vazyme Biotech, Nanjing, China). Briefly, cells were seeded in a six-well plate and cultured for 48 h. Then, the cells were collected and fixed with 70% pre-chilled ethanol overnight at 4 °C. After washing with phosphate-buffered saline (PBS) twice, the cells were stained with 50 μg/mL PI containing PBS–Triton X-100 for 30 min at room temperature. Finally, the stained cells were analyzed by a FACSCalibur flow cytometer (BD Biosciences, New York, NY, USA).

For apoptosis assay, the cells were seeded into 6-well plates and treated for 48 h. The cell apoptosis was examined using an AnnexinV FITC Apoptosis Detection Kit (Vazyme Biotech, Nanjing, China) according to the manufacturer’s instructions. Apoptotic cells were measured by a FACSCalibur flow cytometer (BD Biosciences, New York, NY, USA).

### Transwell assay

Migration and invasion assay was conducted using Transwell chambers (Corning Incorporated, Corning, NY, USA). For migration assay, 4 × 10^4^ cells seeded into a transwell insert containing 5% DMEM serum. After incubation of 24–48 h, cells on the bottom side of the membrane were treated with 95% alcohol and stained with crystal violet for 20 min at room temperature. Next, the number of cell on the lower side was counted using a microscope. For invasion assay, matrix gel was added to the upper surface before the cells were seeded. The remaining steps were the same as above.

### Transient transfection

MiR-122-5p mimics and corresponding negative control (miR-NC), siRNA against CCNG1 (si-CCNG1) and its scrambled control (si-con) were purchased from GenePharma (Shanghai, China). The sequence of miR-122-5p mimics was 5′-AACGCCAUUAUCACACUAAAUA-3′. The sequence of mimics control was 5′-GUGCACGAAGGCUCAUCAUU-3′. The siRNA sequences for CCNG1 were 5′-GGUGUGUUGGAAAGUCAAAGC-3′ (sense) and 5′-UUUGACUUUCCAACACACCUU-3′ (antisense). PDAC cells were seeded into 24-well plates at a density of 2 × 10^5^ cells per well 1 day before transfection. To manipulate the expressions of miR-122-5p or CCNG1, lipofectamine 2000 reagent (Invitrogen, Carlsbad, CA, USA) was used for transfection with 40 nmol/L miR-122-5p mimics and miR-NC or 50 nmol/L si-CCNG1 and si-con according to the manufacturer’s instructions. After a 24–48 h of culture, the cells were further incubated for subsequent experiments.

### Construction of stable cell lines

Following the experimental design, lentiviral vector was used to construct the LV2-hsa-miR-122-5p-mimic vector (L-miR-122-5p-mimic) (GenePharma, Shanghai, China). The structure was used to overexpress miR-122-5p in PC cells after being verified by DNA sequencing. The LV2 empty lentiviral construct (L-miR-NC) served as a negative control. When PANC-1 and PL-45 grew to 50–60% confluence, they were infected by L-miR-122-5p-mimic and L-miR-NC at an appropriate multiplicity of infection (MOI). Stable cell lines were screened by using 5 μg/mL puromycin (Sigma, Aldrich) for a week. The coding sequence of CCNG1 was amplified and cloned into a pcDNA3.1 vector to generate CCNG1 overexpression vector, and the empty pcDNA3.1 vector was used as a control.

### Immunohistochemistry

Tumor tissue sections were incubated in a dry oven at 60 °C for 1 h, then dealkylated in xylene and rehydrated with graded ethanol. Then the slides were pretreated in 0.01 M citrate buffer (pH 6.0) for 10 min in microwave oven, and antigen retrieval was carried out at high power (1200 W). 3% hydrogen peroxide (H2O2) was subsequently used to block the activity of endogenous peroxidase at room temperature for 30 min. After sealing with 5% BSA (bovine serum albumin, Boster Bioengineering, Wuhan, China), the sections were incubated with diluted CCNG1 antibody (1:1000, WH0000900M1, Sigma-Aldrich), Ki-67 antibody (1:400, #9027, Cell Signaling Technology) or Cleaved Caspase-3 antibody (1:250, #9579, Cell Signaling Technology) for 2 h at room temperature. After rinsing, the sections were incubated with the secondary peroxidase conjugated antibody for 45 min at room temperature. Finally, 3, 3-diaminobenzine (DAB, Vector Laboratories, Burlingame, CA, USA) was used for visualization and hematoxylin was used for microscopic examination. The sections were dehydrated, cleared and placed under the light microscope.

### Luciferase reporter assay

The full-length of 3′UTR CCNG1 containing the putative wild-type (wt) miR-122-5p binding sites were amplified by PCR and cloned into pmirGlo luciferase reporter vector (named pmirGlo-CCNG1-WT; WT). QuikChange Multi Site-Directed Mutagenesis kit (Agilent Technologies, Inc., Santa Clara, CA, USA) was used for the site-directed mutagenesis (mut) of CCNG1 3′-UTR (pmirGlo-CCNG1-MUT; MUT) with WT as control. The constructed luciferase reporter plasmids CCNG1-wt or CCNG1-mut were co-transfected with miR-122-5p mimics or negative control into HEK293T cells using Lipofectamine 2000 reagent. After transfection for 48 h, the luciferase activity was determined using a Dual-Luciferase Reporter Assay system (Promega Corporation, Madison, WI, USA) according to the manufacturer’s protocol.

### Tumor xenograft study in vivo

Six-week-old male BALB/c nude mice (weight: 250–350 g) were purchased from the Shanghai Institute for Biological Sciences (Shanghai, China). All animal experiments were carried out in compliance with the guidelines of the Research Ethics Committee of the First Affiliated Hospital of Soochow University. To construct a xenograft tumor model, the mice were randomly divided into miR-122-5p mimic and control groups (n = 5 each). For miR-122-5p mimic group, 1 × 10^7^/0.2 mL PANC-1 cells stably expressing miR-122-5p were subcutaneously injected into the mice. For control group, 1 × 10^7^/0.2 mL PANC-1 cells transfected with L-miR-NC were subcutaneously injected into the mice. After a week of tumor formation, the tumor volume of each mouse was measured at day 6, 9, 12, 15, 18, 21 and 24 after inoculation using Vernier caliper. The volume of tumor was calculated with the formula of V = 0.5*length*width^2. At day 25, the tumors were excised and miR-122-5p expression was analyzed by miRNA RT-PCR. The expression of CCNG1 was measured by qRT-PCR and western blot analysis.

### Statistical analysis

All statistical analyses were performed with SPSS 17.0 software. Every experiment in vitro was performed in triplicate and repeated three times. All data were presented as mean ± standard deviation (SD). Differences between groups were evaluated using unpaired Student’s t-test and one-way ANOVA. P < 0.05 was considered statistically significant.

## Results

### MiR-122-5p expression was downregualted in both PDAC tissues and cell lines

**Fig. 1:**
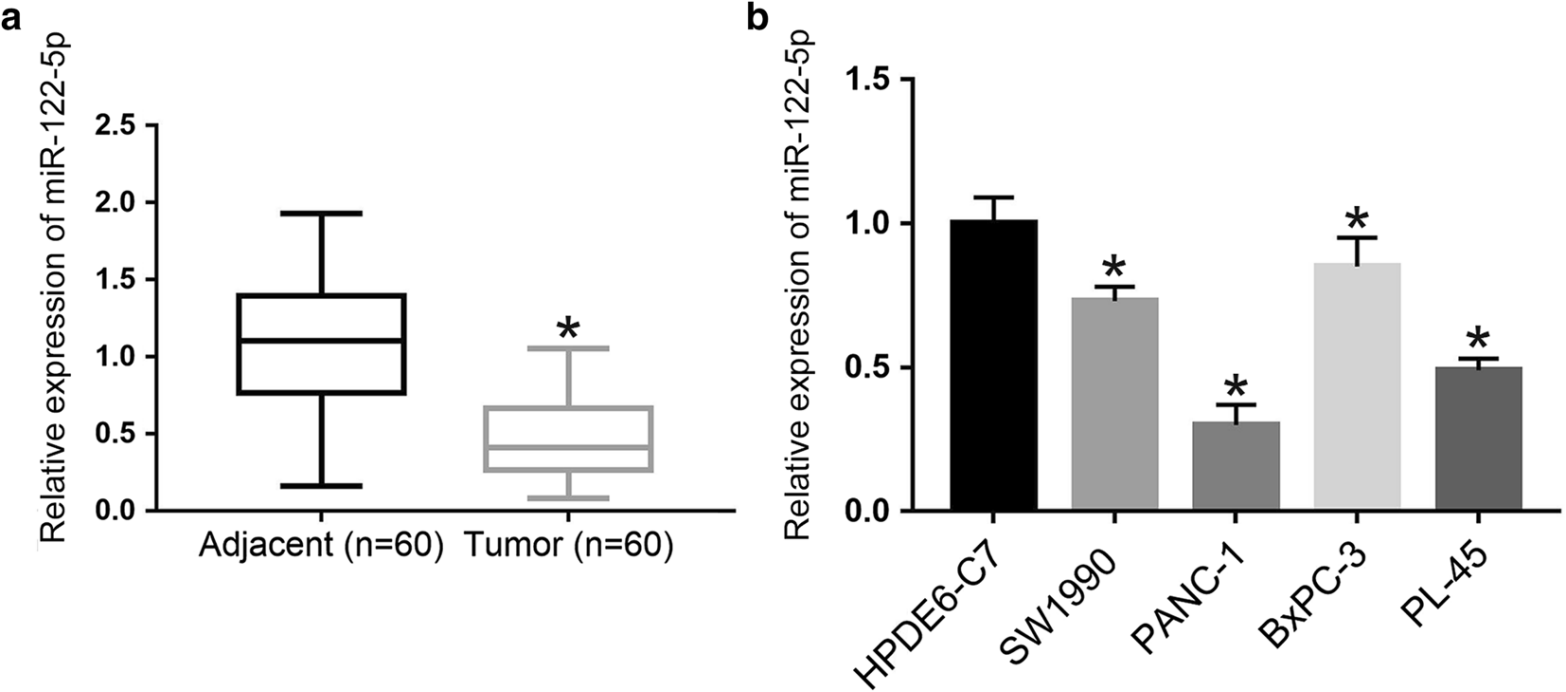
The expression of miR-122-5p in PDAC tissues and cells. **a** The expression levels of miR-122-5p in 60 cases of human PDAC tissues and paired adjacent normal tissues were examined by miRNA RT-PCR. **b** The expression levels of miR-122-5p in PDAC cells and HPDE6-C7 cells were detected using miRNA RT-PCR (*P < 0.05)

To confirm whether miR-122-5p was abnormally regulated in PDAC tissues, 60 pairs of PDAC tissues and matched normal tissues were collected to detect the relative expression of miR-122-5p by miRNA RT-PCR. As shown in Fig. 1a, compared with the paired adjacent normal tissues, the expression of miR-122-5p was downregulated in human PDAC tissues. The miR-122-5p expression was further examined in human normal pancreatic duct epithelial cells (HPDE6-C7) and PDAC cell lines (SW1990, PANC-1, BxPC-3, PL-45) by miRNA RT-PCR. As shown in Fig. 1b, the expression level of miR-122-5p was lower in PDAC cell lines than that in normal cell line. Furthermore, we investigated the correlation between the miR-122-5p expression and clinicopathologic features of PDAC. 60 PDAC patients were divided into a miR-122-5p overexpression group and a miR-122-5p low expression group according to expression levels of miR-122-5p whether higher than the mean expression or not. As shown in Table 1, 27 cases belonged to the high miR-122-5p group, while 33 cases belonged to the low miR-122-5p group. Downregulated miR-122-5p expression was associated with advanced tumor-node-metastasis (TNM) stage, larger tumor size and positive lymph node metastasis. These data indicated that miR-122-5p was downregulated in PDAC tissues and cell lines.

### MiR-122-5p inhibits proliferation, migration and invasion of PDAC cell lines

**Fig. 2:**
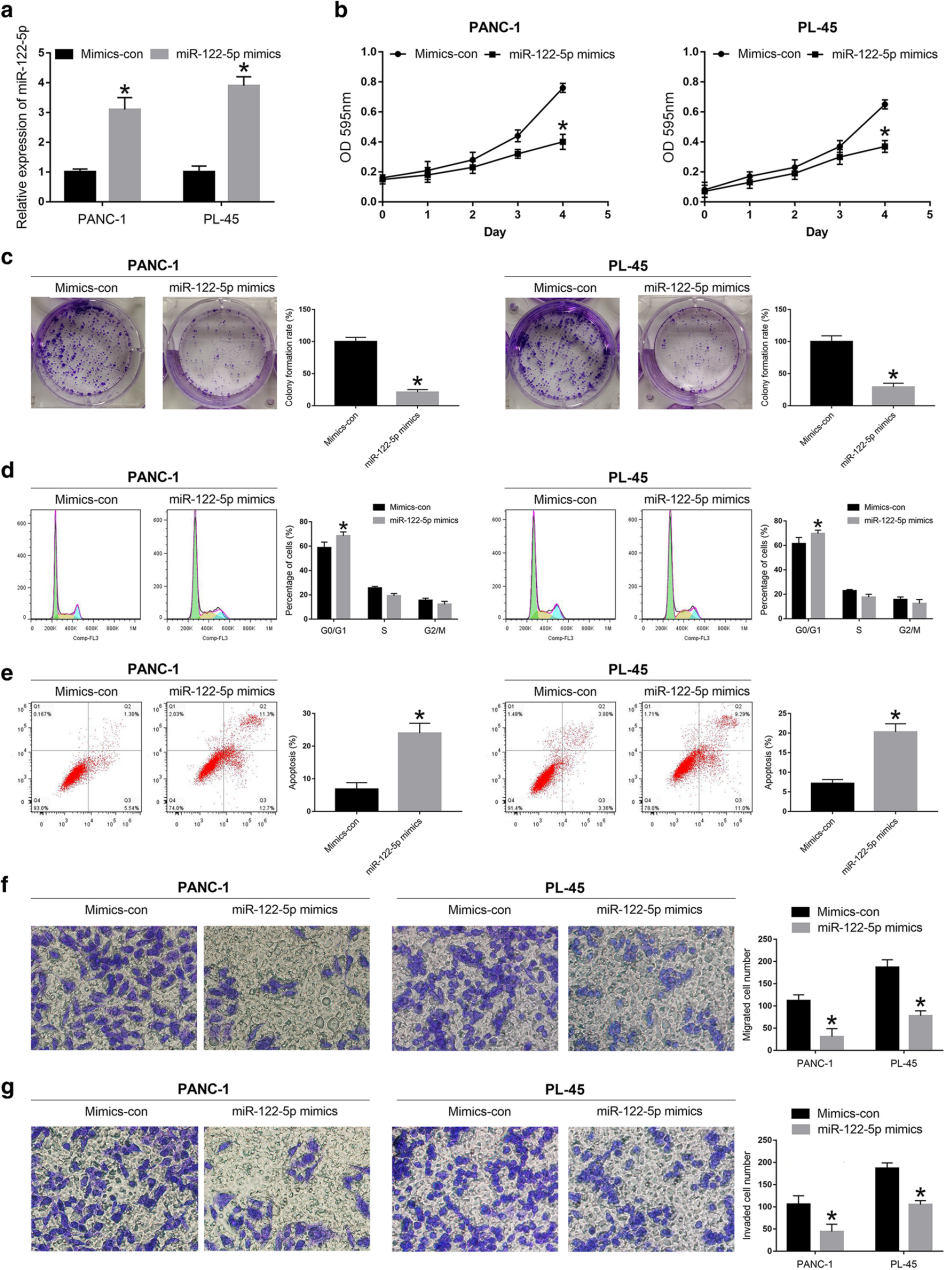
MiR-122-5p inhibited the proliferation, migration and invasion of PDAC cells. **a** The efficacy of transfection was detected in PDAC cells transfected with miR-122-5p mimics. **b** MTT assay was applied to determine the proliferation of PDAC cells transfected with miR-122-5p mimics. **c** Effects of miR-122-5p overexpression on the colony formation of PDAC cells. **d**–**g** Effects of miR-122-5p difference on cell cycle distribution, apoptosis, migration and invasion of PDAC cells (*P < 0.05)

To assess the role of miR-122-5p in PDAC development, we selected PANC-1 and PL-45 cell lines to verify the biological function of miR-122-5p according to the results of miRNA RT-PCR. MiR-122-5p mimics and mimics control were constructed to transfect PANC-1 and PL-45 cells, respectively. MiRNA RT-PCR was applied to detect the efficiency of the transfection. As shown in Fig. 2a, miR-122-5p was remarkably increased in PANC-1-mimics and PL-45-mimics compared to the levels detected in the control groups. MTT assay was performed to verify the effect of miR-122-5p on the proliferation of PDAC cells. The results suggested that proliferation rate of PANC-1 cells transfected with miR-122-5p mimics was significantly decreased compared to that transfected with mimics control (mimics-con) (Fig. 2b). Similar result was observed in PL-45 cells (Fig. 2b). Consistent with the results of MTT assay, colony formation assay revealed that upregulated miR-122-5p could decrease the proliferation in both PANC-1 and PL-45 cell lines (Fig. 2c). To further evaluate whether the inhibitory effect of miR-122-5p on PDAC cells was due to cell cycle arrest and/or apoptotic death, flow cytometry was conducted to reflect the cell cycle distribution and apoptotic rate of PANC-1 and PL-45 cells. The results revealed that both PANC-1 and PL-45 cells that transfected with miR-122-5p mimics extended cell cycle in the G0/G1 phase (Fig. 2d). In PANC-1 cells, increase in the percentage of cells in G0/G1 phase was from 58.9 ± 4.5% to 68.6 ± 3.1%, with concomitant decrease in the percentage of cells in S phase from 25.7 ± 1.2% to 19.2 ± 1.9% and G2/M phase from 15.4 ± 1.8% to 12.2 ± 2.4%. In PL-45 cells, increase in the percentage of cells in G0/G1 phase was from 61.5 ± 5.1% to 69.7 ± 2.8%, with concomitant decrease in the percentage of cells in S phase from 22.8 ± 1.1% to 17.7 ± 2.3% and G2/M phase from 15.7 ± 2.1% to 12.6 ± 3.2%. In addition, compared with mimics-con groups, the miR-122-5p mimics groups significantly prompted apoptosis in PANC-1 and PL-45 cells (Fig. 2e). In summary, these data mentioned above demonstrated that upregulation of miR-122-5p could inhibit proliferation of PDAC cells through inducing cell cycle arrest at G0/G1 phase and prompting apoptosis.

**Fig. 3:**
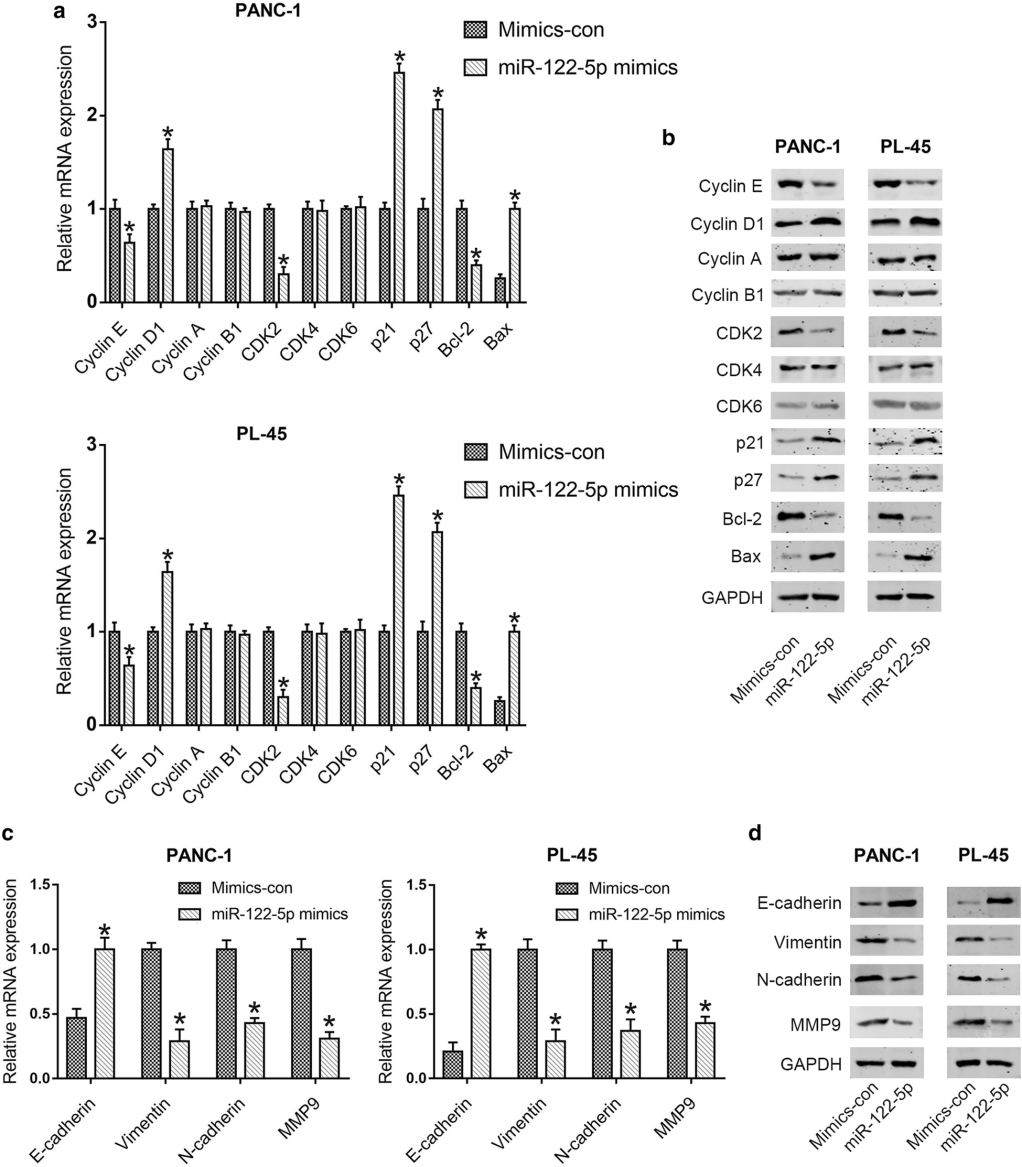
MiR-122-5p regulated the expression of proteins associated with cell cycle and apoptosis as well as migration and invasion. **a**, **b** The expression levels associated with cell cycle and apoptosis were determined in PDAC cells using qRT-PCR and western blot after transfection with miR-122-5p mimics. **c**, **d** The expression levels associated with migration and invasion were examined in PDAC cells transfected with miR-122-5p mimics by qRT-PCR and western blot (*P < 0.05)

The effects of miR-122-5p on migration and invasion of PDAC cell lines were investigated by transwell assay. Compared with the control cells, the number of migrated cells was significantly decreased by highly expressed miR-122-5p in PANC-1 and PL-45 cells (Fig. 2f). In the invasion assay, increased miR-122-5p expression inhibited the number of invaded cells of PDAC cell lines (Fig. 2g). These data indicated that miR-122-5p suppressed cell migration and invasion in PDAC cells. In addition, the expression levels of molecules correlated with cell cycle and apoptosis (Cyclin E, Cyclin D1, Cyclin A, Cyclin B1, CDK2, CDK4, CDK6, p21, p27, Bcl-2 and Bax), as well as EMT (E-cadherin, Vimentin, N-cadherin and MMP9), were detected by qRT-PCR and western blot analysis. As shown in Fig. 3a, b, the expression of Cyclin E, CDK2 and Bcl-2 was significantly reduced, while the expression of Cyclin D1, p21, p27 and Bax was elevated at mRNA and protein levels in mimics groups compared with that in control groups. There were no significant changes in the expression levels of Cyclin A, Cyclin B1, CDK4 and CDK6. Furthermore, compared to the control groups, increased expression of E-cadherin and decreased expression of Vimentin, N-cadherin and MMP9 were observed in mimics groups (Fig. 3c, d).

### CCNG1 is a target gene of miR-122-5p and up-regulated in human PDAC tissues and cell lines

**Fig. 4:**
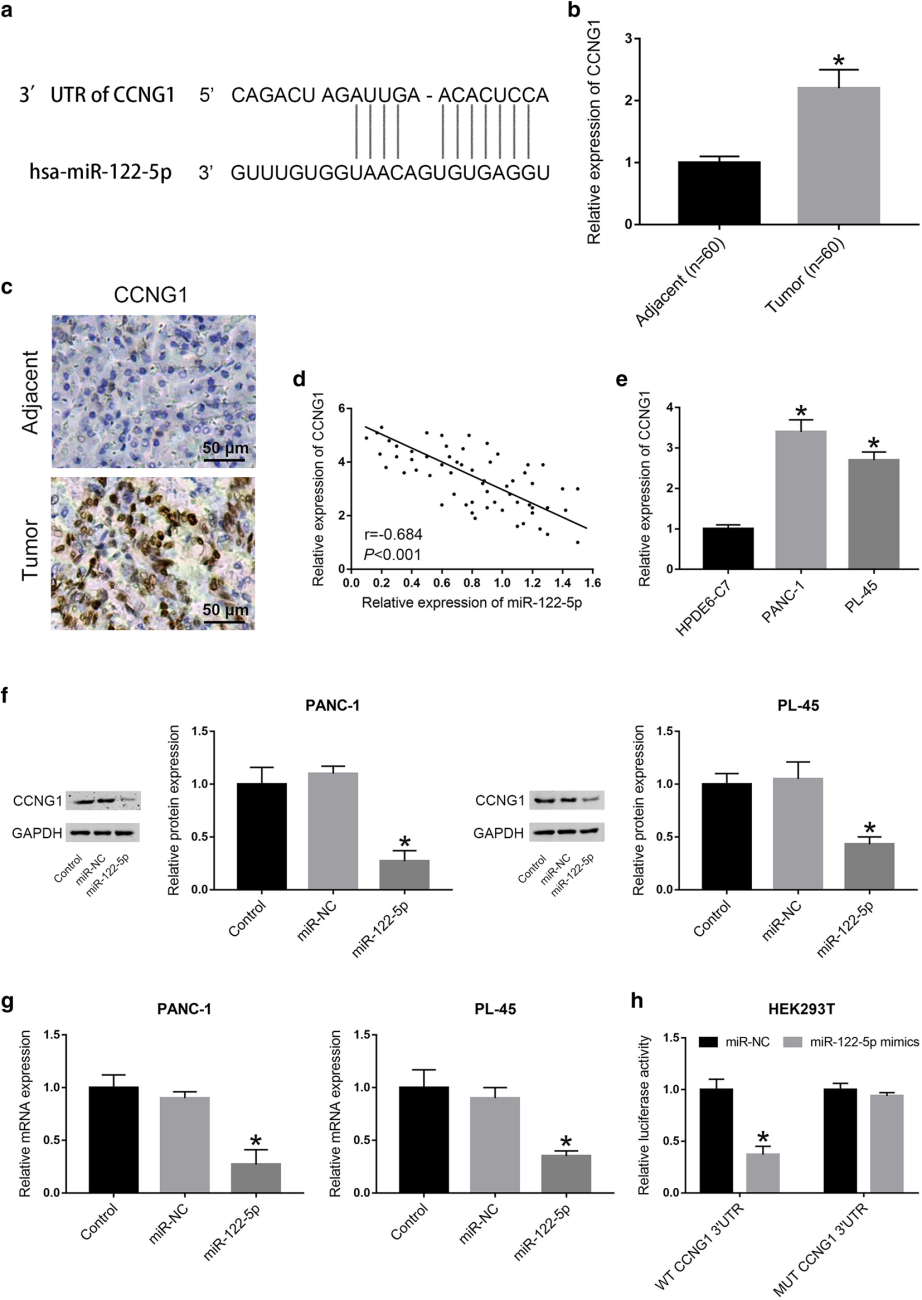
CCNG1 was upregulated in PDAC tissues and cells and was verified to be a direct target of miR-122-5p. **a** The predicted target site of miR-122-5p on 3′-UTR of CCNG1 mRNA. **b** 60 pairs of human PDAC tissues and paired normal tissues were collected to detect the relative expression of CCNG1 by qRT-PCR. **c** Immunohistochemistry analysis of CCNG1 expression in human PDAC tissue and paired normal tissue. **d** The correlation between the expression levels of miR-122-5p and CCNG1. **e** The relative expression of CCNG1 was determined in PDAC cells compared to HPDE6-C7 cells. **f**, **g** Effects of highly expressed miR-122-5p on the expression of CCNG1 in PDAC cells were verified by western blot and qRT-PCR. **h** Effects of miR-122-5p overexpression on the luciferase activity in HEK293T cells (*P < 0.05)

To predict the target genes of miR-122-5p, Targetscan (http://www.targetscan.org/vert_71/), PicTar (https://pictar.mdc-berlin.de/) and miRDB (http://www.mirdb.org) were applied. According to the prediction results from the miRNA bioinformatics websites, we found that CCNG1, an oncogene in many human cancers, might be a target gene of miR-122-5p (Fig. 4a). To assess the correlation of miR-122-5p and CCNG1 in PDAC, 60 pairs of PDAC tissues and matched adjacent normal tissues were collected to detect the expression level of CCNG1 by qRT-PCR. The results showed that the expression level of CCNG1 in PDAC tissues was higher than that in normal tissues (Fig. 4b). To further explore the expression of CCNG1 in PDAC, we investigated the reactivity for CCNG1 by immunohistochemical staining. Representative example of reactivity for CCNG1 was shown (Fig. 4c). Interestingly, elevated expression of CCNG1 was observed in PDAC tissue compared with that in adjacent normal tissue. Notably, there was a negative correlation between the expression levels of miR-122-5p and CCNG1 (Fig. 4d). Next, CCNG1 expression level was detected in PDAC cells and HPDE6-C7 using qRT-PCR. The data demonstrated that the expression of CCNG1 was upregulated in PDAC cells compared to HPDE6-C7 cells (Fig. 4e). To verify the mechanism by which miR-122-5p regulated CCNG1, western blot and qRT-PCR were applied to evaluate CCNG1 expression levels after transfection with miR-122-5p mimics. The CCNG1 protein expression was significantly decreased in both PANC-1 and PL-45 cells after transfected with miR-122-5p mimics compared to the control group (Fig. 4f). Consistent with the results from western blot, the CCNG1 mRNA expression levels in PDAC cells transfected with miR-122-5p mimics were evidently lower than that in control group (Fig. 4g). To determine whether miR-122-5p directly targets CCNG1, a dual-luciferase reporter system containing the wild-type (WT) and mutant-type (MUT) 3′-UTR of CCNG1 was employed. HEK293T cells were co-transfected with reporter plasmids and pre-miR-122-5p or the pmirGLo empty vector (control). Cells that transfected with pre-miR-122-5p/WT-CCNG1-UTR exhibited a remarkable decrease in luciferase activity. Instead, overexpression of miR-122-5p did not affect luciferase activity in the cells transfected with pre-miR-122-5p/MUT-CCNG1-UTR (Fig. 4h). The results suggested that miR-122-5p directly targets the 3′-UTR of CCNG1.

### Downregulation of CCNG1 inhibits proliferation, migration and invasion in PDAC cells

**Fig. 5:**
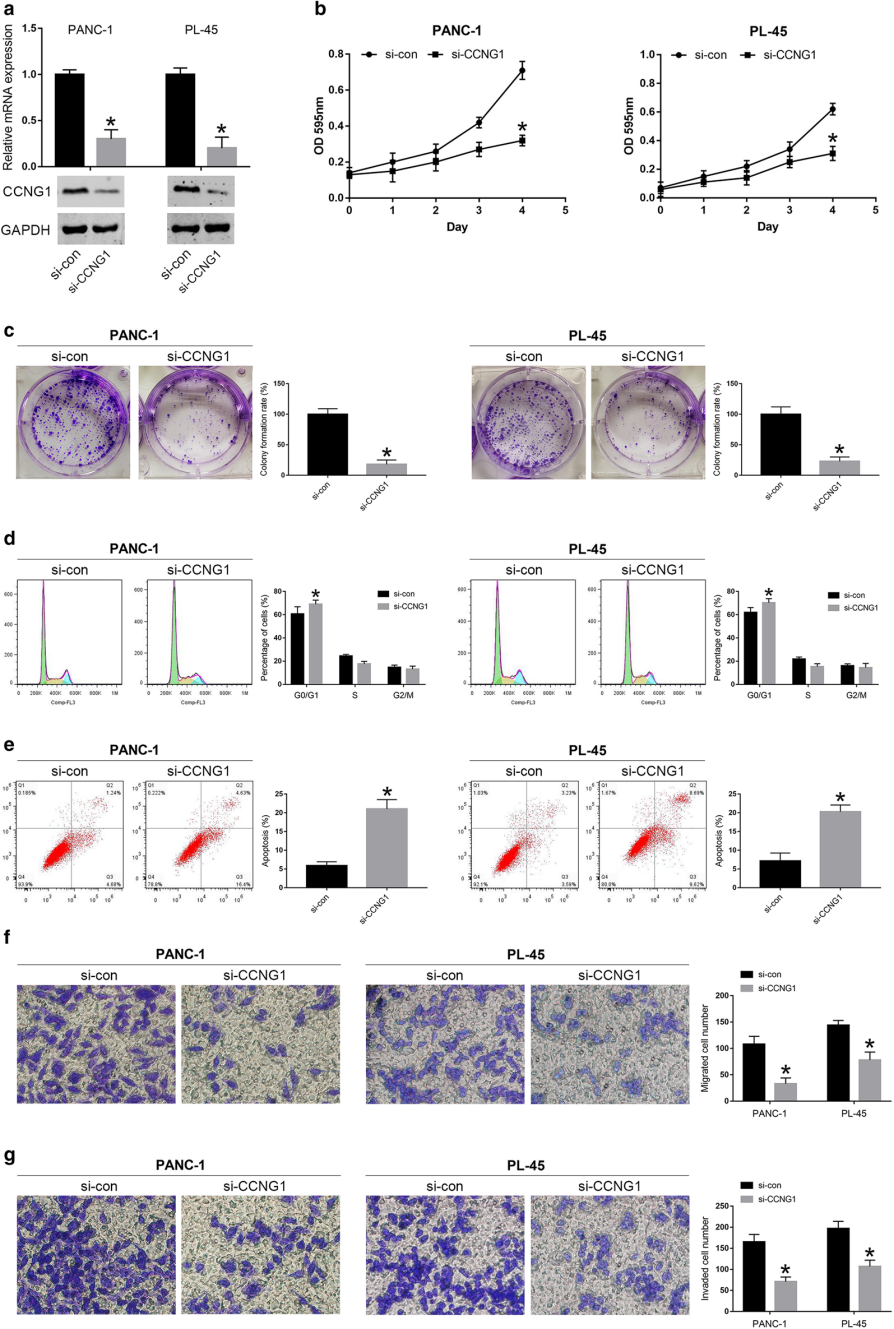
Silencing of CCNG1 expression inhibited the proliferation, migration and invasion of PDAC cells. **a** Efficacy of PDAC cells transfected with si-CCNG1 on CCNG1 expression was confirmed by qRT-PCR and western blot. **b** The cell viability of PDAC cells transfected with si-CCNG1 was detected using MTT assay. **c** The colony formation of transfected PANC-1 and PL-45 cells was examined. **d**–**g** Effects of CCNG1 difference on cell cycle distribution, apoptosis, migration and invasion of PDAC cells (*P < 0.05)

**Fig. 6:**
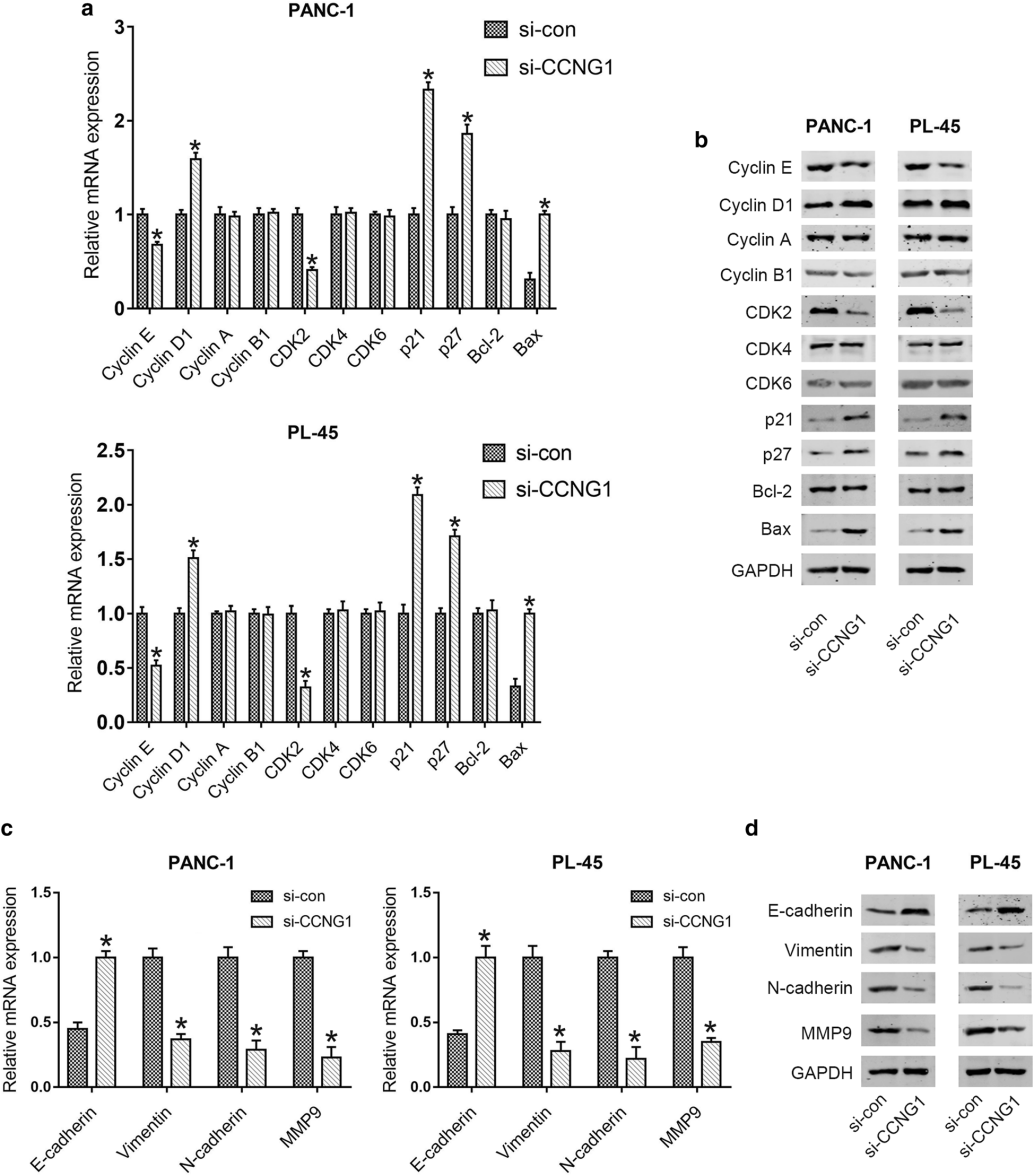
Knock down of CCNG1 influenced the expression levels of proteins associated with cell cycle and apoptosis as well as migration and invasion. **a**, **b** The expression levels associated with cell cycle and apoptosis were detected in PDAC cells by qRT-PCR and western blot after transfection with si-CCNG1. **c**, **d** The expression levels associated with migration and invasion were determined in PDAC cells transfected with si-CCNG1 by qRT-PCR and western blot (*P < 0.05)

To further explore the potential mechanism by which miR-122-5p regulated the cell behavior of human PDAC cells, we assessed the function of CCNG1 in PANC-1 and PL-45 cells by downregulation of the gene. As shown in Fig. 5a, we observed a significantly downregulated expression of CCNG1 at both mRNA and protein levels in PDAC cells that transfected with si-CCNG1. When compared with si-con group, si-CCNG1 group exhibited significantly less viable cells, less numbers of clone formation, a higher percentage of G0/G1 cells and a lower percentage of S and G2/M cells, as well as a higher proportion of apoptosis and less numbers of migrated and invaded cell (Fig. 5b–g). In PANC-1 cells, increase in the percentage of cells in G0/G1 phase was from 60.7 ± 6.1% to 69.1 ± 3.5%, with concomitant decrease in the percentage of cells in S phase from 24.5 ± 1.3% to 17.7 ± 2.1% and G2/M phase from 14.8 ± 1.9% to 13.2 ± 2.6%. In PL-45 cells, increase in the percentage of cells in G0/G1 phase was from 62.1 ± 4.1% to 70.3 ± 3.5%, with concomitant decrease in the percentage of cells in S phase from 21.8 ± 1.7% to 15.3 ± 2.5% and G2/M phase from 16.1 ± 1.6% to 14.4 ± 3.7%. In addition, lower expression of Cyclin E and CDK2 and higher expression of Cyclin D1, p21, p27 and Bax were observed at mRNA and protein levels in PDAC cells after transfection with siCCNG1 compared to the control group (Fig. 6a, b). However, no significant changes were observed in the expression of Cyclin A, Cyclin B1, CDK4, CDK6 and Bcl-2 (Fig. 6a, b). For EMT associated molecules, we found that the expression of E-cadherin was increased, while the expression of Vimentin, N-cadherin and MMP9 was reduced in si-CCNG1 group compared to control group (Fig. 6c, d).

### MiR-122-5p inhibits proliferation, migration and invasion of PDAC cells via targeting CCNG1

**Fig. 7:**
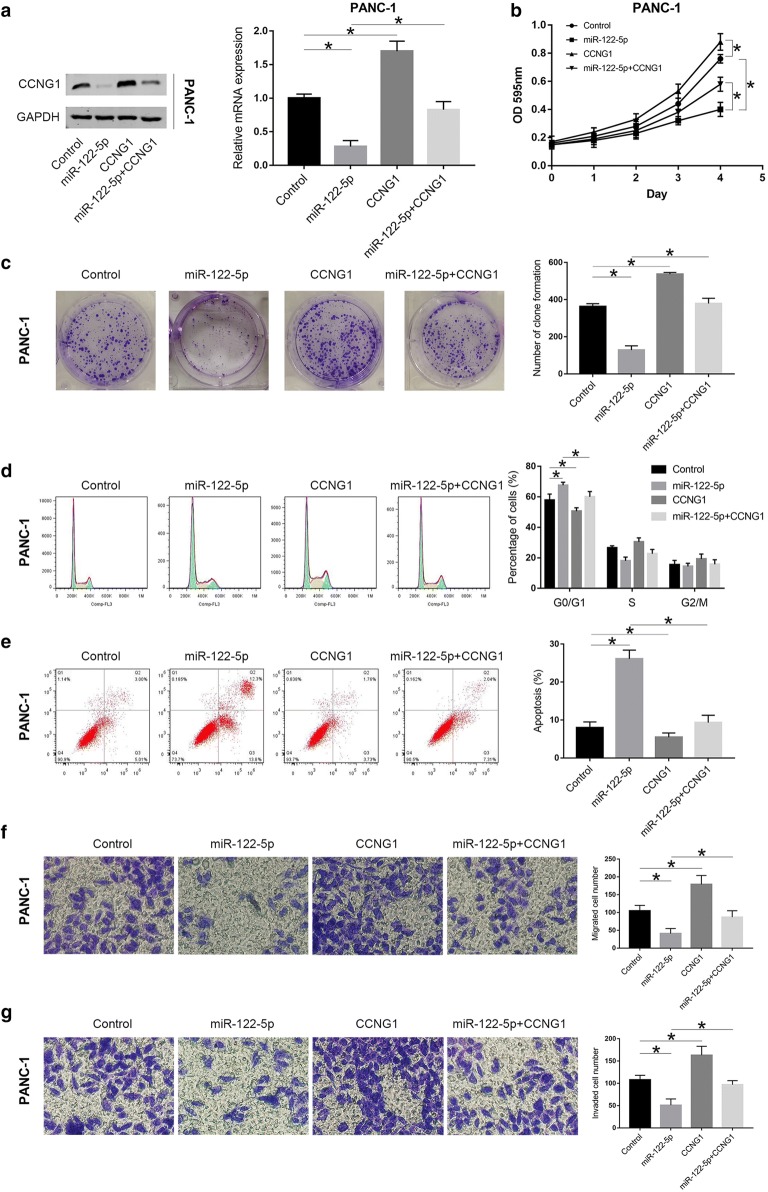
Highly expressed CCNG1 could partially reverse the effects of miR-122-5p on PANC-1 cells. **a** Western blot and qRT-PCR were conducted to confirm the expression of CCNG1 in each group. **b** MTT assay was performed to verify the effect of ectopic CCNG1 expression on cell viability induced by miR-122-5p overexpression in PANC-1 cells. **c** Colony formation of transfected PANC-1 cells was detected. **d**–**g** The effects of CCNG1 alteration in cell cycle distribution, apoptosis, migration and invasion of PANC-1 cells were confirmed (*P < 0.05)

To further determine whether miR-122-5p inhibited proliferation, migration and invasion in PDAC by directly regulating CCNG1, the coding sequence of CCNG1 was cloned into a pcDNA3.1 vector to generate CCNG1 overexpression vector. Panc-1 and PL-45 cells were next co-transfected with miR-NC + CCNG1-NC, miR-122-5p mimics + CCNG1-NC, miR-NC + CCNG1, and miR-122-5p mimics + CCNG1. Western blot and qRT-PCR were applied to detect CCNG1 expression at protein and mRNA levels in both cell lines (Fig. 7a and Additional file 1: Fig. S1a). Through MTT assay, colony formation assay, flow cytometry analysis and transwell assay, we found that ectopic CCNG1 expression could reverse the suppression of proliferation, migration and invasion induced by miR-122-5p overexpression in PANC-1 and PL-45 cells (Fig. 7b–g and Additional file 1: Fig. S1b–g). For mimics + CCNG1-NC vs mimics + CCNG1group, decrease in the percentage of cells in G0/G1 phase was from 67.6 ± 1.9% to 60.1 ± 3.5%, with concomitant increase in the percentage of cells in S phase from 18.0 ± 2.5% to 22.7 ± 2.8% and G2/M phase from 14.4 ± 2.1% to 17.2 ± 3.0% in PANC-1 cells, and decrease in the percentage of cells in G0/G1 phase was from 69.2 ± 2.1% to 62.9 ± 2.5%, with concomitant increase in the percentage of cells in S phase from 19.5 ± 1.6% to 23.8 ± 2.1% and G2/M phase from 11.3 ± 1.9% to 13.3 ± 1.8% in PL-45 cells. These observations indicated that miR-122-5p played an inhibitory effect on proliferation, migration and invasion of PDAC cells by directly targeting CCNG1.

### MiR-122-5p inhibits tumorigenicity in vivo

**Fig. 8:**
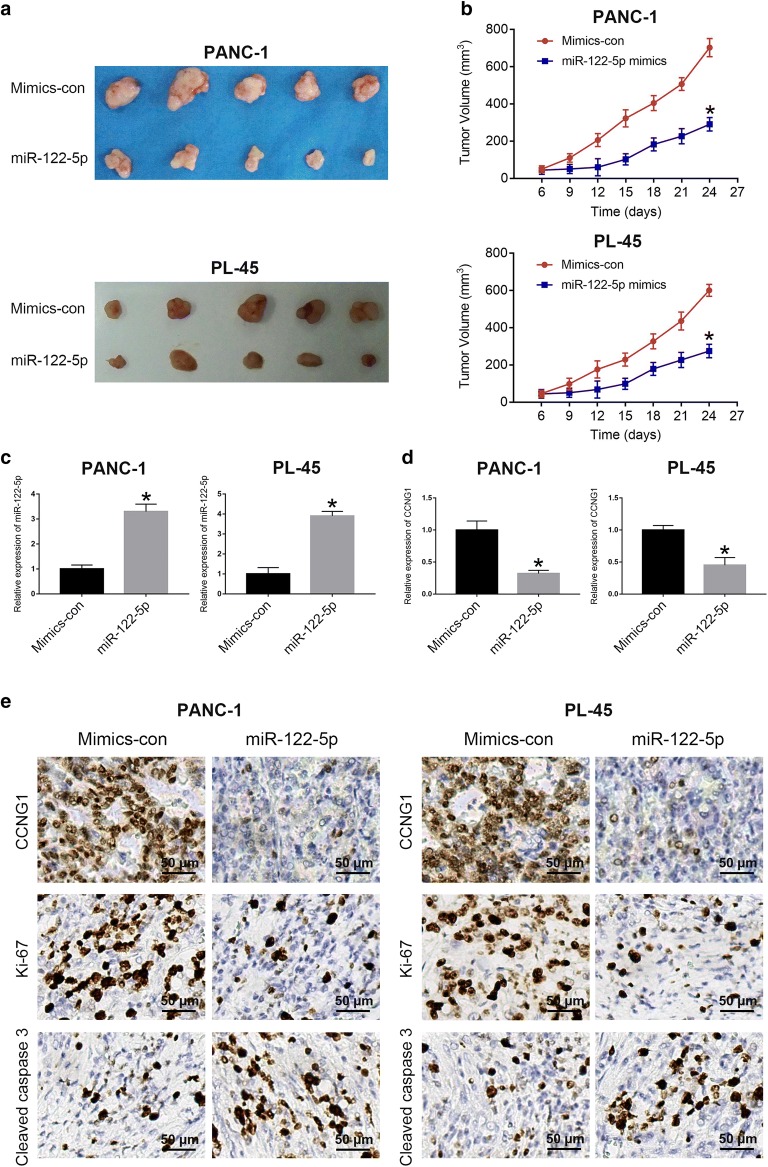
miR-122-5p inhibited tumorigenicity in vivo. **a** Xenograft tumors were obtained from 2 groups of nude mice transfected with mimics control and miR-122-5p mimics respectively. **b** Tumor growth curve was significantly different between miR-122-5p mimics and mimics control group. **c** The expression of miR-122-5p in the implanted tumors was explored by miRNA RT-PCR. **d** The expression of CCNG1 mRNA level was determined by qRT-PCR. **e** Immunohistochemical staining of CCNG1, Ki-67 and Cleaved caspase 3 in xenograft tumors (*P < 0.05)

To further confirm the role of miR-122-5p in tumorigenicity of PDAC cells in vivo, PANC-1 and PL-45 cell lines stably upregulating miR-122-5p were injected into the right flank of nude mice to generate tumor ectopically. As shown in Fig. 8a, b, mice injected with miR-122-5p overexpression PANC-1 cells or PL-45 cells had smaller tumor volume compared with those in the negative control group. Furthermore, miRNA RT-PCR demonstrated that miR-122-5p expression dramatically increased in miR-122-5p overexpression group compared with that in the negative control group (Fig. 8c). Reversely, CCNG1 expression was evidently reduced in miR-122-5p overexpression group (Fig. 8d). Moreover, immunohistochemical analysis of resected tumor demonstrated that miR-122-5p mimics group had significantly decreased CCNG1 and Ki-67, but increased Cleaved caspase 3 (Fig. 8e). These findings suggested that miR-122-5p suppressed tumor formation in vivo.

## Discussion

In recent years, significant advances in miRNA research have provided valuable clues for elucidating the occurrence and progression of cancers [22]. Analysis of the aberrant expression of miRNAs in cancer tissue samples or cell lines has provided important information for indentifying tumor therapeutic targets [23]. It has been reported that miRNA could act as an oncogene or tumor suppressor in cancers via directly targeting mRNA6. Previous researches have demonstrated that miR-122-5p is aberrantly expressed and plays significant roles in different human malignancies [11–13]. However, the biological function of miR-122-5p and its potential molecular mechanism in PDAC remain to be elucidated. In the current study, miR-122-5p expression was significantly decreased in PDAC tissues and cell lines compared to paired normal adjacent tissues and HPDE6-C7 cells. Moreover, we found that there was a negatively correlation between miR-122-5p expression and clinicopathological features. The results showed that downregulated miR-122-5p expression was associated with advanced TNM stage, larger tumor size and positive lymph node metastasis. Furthermore, on the basis of a series of experiments, overexpression of miR-122-5p inhibited proliferation, migration and invasion in vitro, and tumorigenicity in vivo. These results indicated that miR-122-5p may be a potential prognostic factor and therapeutic target for PDAC patients.

To elucidate the molecular mechanisms underlying the involvement of miR-122-5p in the proliferation, migration and invasion of PDAC, bioinformatics analysis was employed to predict putative targets of miR-122-5p. Among the candidate target genes, CCNG1 was selected for further study. CCNG1 is a member of G-type cyclins located at chromosome 5q-32-q34, which constitutes of 259 amino acids [24, 25]. It has been reported that CCNG1 plays an important role in the tumorigenesis and progression of many human malignancies [18]. Previous researches have demonstrated that dysregulated CCNG1 mainly exerted a facilitator on various cancers, by promoting epithelial-to-mesenchymal transition (EMT) in esophageal squamous cell carcinoma [21], facilitating migration and invasion in ovarian cancer [19] and stimulating proliferation and metastasis in prostate cancer [26] and lung cancer [20]. In this work, we detected the expression levels of CCNG1 between PDAC tissues and paired adjacent normal tissues, as well as in PDAC cell lines and HPDE6-C7 cells. We found that the expression levels of CCNG1 in PDAC tissues and cell lines were much higher than that in paired normal tissues and HPDE6-C7 cells respectively, which revealed a significantly inverse correlation between CCNG1 expression and miR-122-5p expression in PDAC tissues. Furthermore, the dual-luciferase reporter assay revealed that miR-122-5p directly targeted CCNG1 by recognizing the 3′-UTR of CCNG1 mRNA and overexpression of miR-122-5p could inhibit CCNG1 expression by degrading CCNG1 mRNA. The results also suggested that silencing of CCNG1 expression suppressed cell proliferation by inducing G0/G1 phase arrest and cell apoptosis, as well as migration and invasion. Moreover, ectopic expression of CCNG1 partially counteracted the effects on the suppression of proliferation, migration and invasion caused by miR-122-5p overexpression. These results further verified that the inhibitory effects of miR-122-5p in PDAC were mediated by the downregulation of CCNG1.

It has been reported that there are a serial of genes regulating proliferation. To confirm the accumulation of cells in G0/G1 phase, we examined the expression of Cyclins, CDKs (cyclin-dependent kinases) and CDK inhibitors in the two cell lines. Cyclin is a kind of cell cycle oscillating protein, which is repeatedly expressed and degraded in the whole cell cycle [27]. Cyclin E, Cyclin D1, Cyclin A and Cyclin B1 occur in G1, late G1 or early S, and the S or G2 phases, respectively [28]. Cyclins function by binding CDKs, which play an important role in cell cycle progression of cancer [29]. CDK inhibitor plays the role of ‘cell cycle brake’ which combines the complex of Cyclin and CDK and inhibits their cell cycle accelerator function [30]. In this study, we detected two CDK inhibitors, p21 and p27, both of which are closely related to DNA damage repair and cell cycle arrest [31]. The anti-apoptotic Bcl-2 and pro-apoptotic Bax are pivotal regulators of cell apoptosis [32].

In the present study, we examined the expression levels of Cyclin E, Cyclin D1, Cyclin A, Cyclin B1, CDK2, CDK4, CDK6, p21, p27, Bcl-2 and Bax by qRT-PCR and western blot analysis. The results revealed that PDAC cells transfected with miR-122-5p mimics exhibited significantly reduced expression of Cyclin E, CDK2 and Bcl-2, but increased expression of Cyclin D1, p21, p27 and Bax when compared with control groups. However, no significant changes were observed in the expression levels of Cyclin A, Cyclin B1, CDK4 and CDK6. In addition, PDAC cells transfected with si-CCNG1 showed similar results. However, no significant alterations in the expression of Bcl-2 were observed in PDAC cells transfected with si-CCNG1 compared with control groups, suggesting that the effect of Bcl-2 alteration was induced by miR-122-5p rather than directly by CCNG1. Moreover, Cyclin D1 has been recognized as an oncogene, and its overexpression can promote the proliferation of tumor cells [33]. Interestingly, the expression of Cyclin D1 was increased in PDAC cells transfected with miR-122-5p mimics or si-CCNG1, which was contrary to the inhibitory effect of miR-122-5p on cell cycle. MiR-122-5p overexpression or CCNG1 silencing had modest effects on cell proliferation of the 2 cell lines tested. However, the expression levels of CCNG1 were dramatically decreased upon miR-122-5p upregulation or CCNG1 silencing, suggesting this was not due to incomplete CCNG1 downregulation. The above results suggested that this could be due to compensatory mechanisms by upregulated Cyclin D1 that rescued the inhibitory effects on cell growth. These results provided important clues for the molecular mechanism by which miR-122-5p induced G0/G1 phase arrest and apoptosis in PDAC.

Epithelial–mesenchymal transition (EMT) is a biological process that epithelial cells are transformed into stromal phenotype cells [34]. During this process, the epithelial cells gradually lose their adhesion with the basement membrane, and gain migratory and invasive features [35]. Furthermore, EMT is characterized by reduced expression of cell adhesion proteins, such as E-cadherin, as well as increased expression of mesenchymal associated proteins, such as N-cadherin and Vimentin [36]. It has been known that EMT plays an important role in cancer stemness, metastasis and invasion [37]. In addition, previous studies have reported that miRNAs are closely related to EMT in various human malignancies. A research from Cai et al. found that highly expressed miR-539 significantly suppressed EMT via targeting specificity protein 1 (SP1) in breast cancer [38]. Xiao et al. demonstrated that miR-21 could promote invasion and metastasis of gastric cancer by EMT [39]. In the present study, we examined the expression of EMT associated molecules of PDAC cells after transfection with miR-122-5p mimics or si-CCNG1 according to qRT-PCR and western blot analysis. The results showed that overexpression of miR-122-5p or silencing of CCNG1 could suppress EMT by dramatically increasing the expression of E-cadherin, while decreasing the expression of Vimentin, N-cadherin and MMP9. These findings were consistent with the migration and invasion results in vitro.

**Fig. 9:**
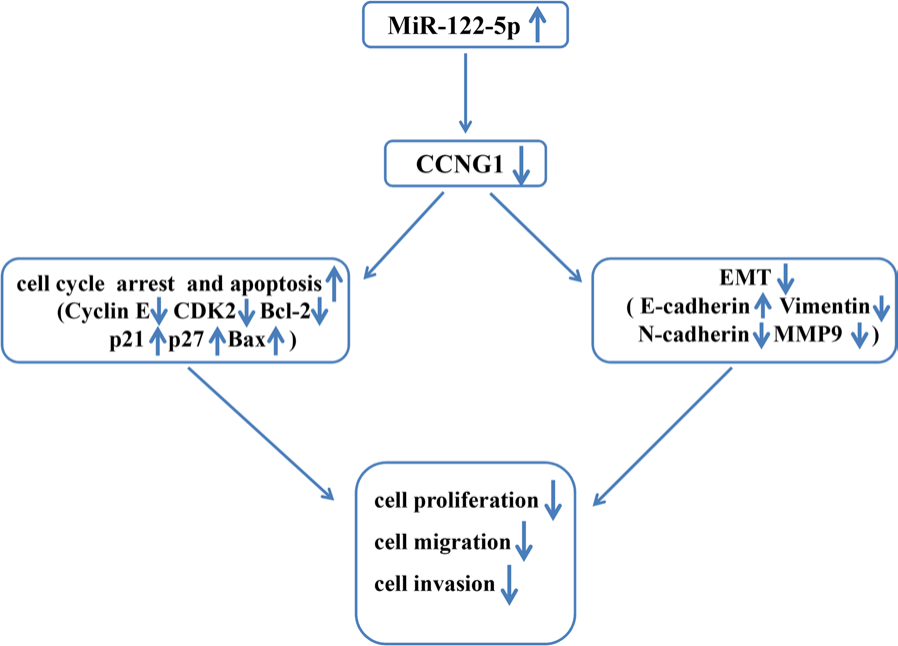
Schematic representation of proposed mechanism of action of miR-122-5p induced G0/G1 phase arrest and apoptosis as well as inhibition of migration and invasion in pancreatic cancer cells. Upregulation of miR-122-5p caused G0/G1 phase arrest by decreasing the levels of Cyclin E and CDK2 and enhancing the expression of p21 and p27 in both cell lines. MiR-122-5p also leaded to apoptosis via Bcl-2 and Bax. Overexpression of miR-122-5p could suppress EMT by dramatically increasing the expression of E-cadherin, while decreasing the expression of Vimentin, N-cadherin and MMP9. Furthermore, miR-122-5p exerted anticancer effects by directly targeting CCNG1

In summary, our results revealed that miR-122-5p was downregulated in PDAC. MiR-122-5p was negatively correlated with TNM stage, tumor size, lymph node metastasis and CCNG1. Overexpression of miR-122-5p suppressed PDAC proliferation, migration, invasion and EMT. Furthermore, miR-122-5p regulated CCNG1 expression via directly binding to the 3′-UTR of CCNG1. Ectopic expression of CCNG1 could counteract the inhibitory effect induced by miR-122-5p overexpression. In general, our findings suggested the miR-122-5p could be a potential therapeutic target for PDAC. According to above results we herein proposed a model for potential mechanism of action of miR-122-5p in pancreatic cancer cells as shown in Fig. 9.

## Conclusions

Our study provides strong evidence for a correlation between miR-122-5p and PDAC. MiR-122-5p suppressed the growth, invasion and migration of PDAC cells by directly targeting CCNG1. Therefore, miR-122-5p may be a promising therapeutic target for PDAC.

## Supplementary information

**Additional file 1: Fig. S1.** Highly expressed CCNG1 could partially reverse the effects of miR-122-5p on PL-45 cells. **a** Western blot and qRT-PCR were conducted to confirm the expression of CCNG1 in each group. **b** MTT assay was performed to verify the effect of ectopic CCNG1 expression on cell viability induced by miR-122-5p overexpression in PL-45 cells. **c** Colony formation of transfected PL-45 cells was detected. **d**–**g** The effects of CCNG1 alteration in cell cycle distribution, apoptosis, migration and invasion of PL-45 cells were confirmed (*P < 0.05).

## Data Availability

All data generated or analyzed during this study are included in this published article.

## Abbreviations

PDAC: Pancreatic ductal adenocarcinoma
miRNAs: MicroRNAs
CCNG1: Cyclin G1
UTR: Untranslated regions
RPMI: Roswell Park Memorial Institute
DMEM: Dulbecco’s modified Eagle’s medium
cDNA: Complementary DNA
MTT: 3- (4, 5-Dymethyl-2-thiazolyl)-2, 5-diphenyl-2H-tetrazolium bromide
SDS-PAGE: Sodium dodecyl sulfate polyacrylamide gel electrophoresis
PI: Propidium iodide
BSA: Bovine serum albumin
EMT: Epithelial–mesenchymal transition
RT-PCR: Quantitative reverse transcriptionreal-time polymerase chain reaction
MMP: Matrix metalloproteinase
ANOVA: Analysis of variance
