# Supplementary figures and images for: MicroRNA-122-5p inhibits cell proliferation, migration and invasion by targeting CCNG1 in pancreatic ductal adenocarcinoma
Source: Cancer Cell Int. 2020 Mar 30;20:98. doi: 10.1186/s12935-020-01185-z (PMC7106816; doi:10.1186/s12935-020-01185-z)

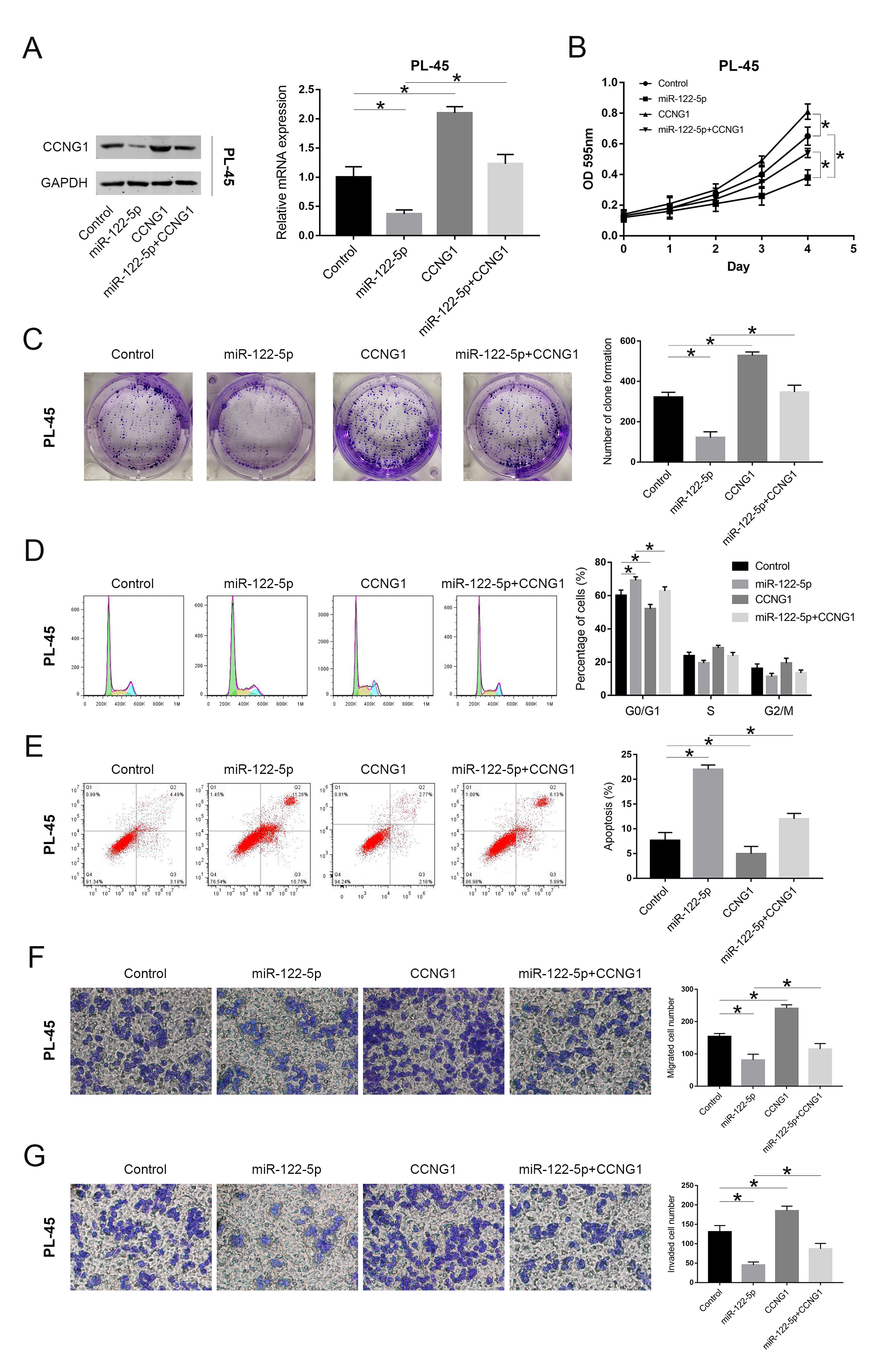

Supplement: Supplementary file 1 — Additional file 1: Fig. S1. Highly expressed CCNG1 could partially reverse the effects of miR-122-5p on PL-45 cells. a Western blot and qRT-PCR were conducted to confirm the expression of CCNG1 in each group. b MTT assay was performed to verify the effect of ectopic CCNG1 expression on cell viability induced by miR-122-5p overexpression in PL-45 cells. c Colony formation of transfected PL-45 cells was detected. d–g The effects of CCNG1 alteration in cell cycle distribution, apoptosis, migration and invasion of PL-45 cells were confirmed (*P < 0.05). [file 12935_2020_1185_MOESM1_ESM.tif]
